# How sport changed my life? Description of the perceived effects of the experiences of young Colombians throughout a sport for development and peace program

**DOI:** 10.3389/fspor.2023.1046937

**Published:** 2023-05-04

**Authors:** Tegwen Gadais, Natalia Varela Pulido, Victoria Soto, Sandra Vinazco, Mauricio Garzon

**Affiliations:** ^1^ Département des Sciences de l’activité physique, Université du Québec à Montréal, Montréal, QC, Canada; ^2^ Chaire UNESCO en Développement Curriculaire (CUDC), Université du Québec à Montréal, Montréal, QC, Canada; ^3^ Facultad de Ciencias Sociales y Humanas, Universidad Externado de Colombia, Bogotá, Colombia; ^4^ Deutsche Sport Schule Köln, Köln, Germany

**Keywords:** program, perceptions, sport for peace, sport for development, Latin America and Caribbean, Colombia

## Abstract

**Introduction:**

This study contributes to the advancement of the field of Sport for Development and Peace (SDP) research in Latin America and the Caribbean (LAC). There are still few studies on SDP programs in this region and it is important to document and understand the impacts of these programs on participants.

**Methods:**

The present study is the result of a collaborative research that aims to describe the experiences and perceptions of Colombian youth and program managers who participated in an SDP program that took them from a local community sports club to the Olympic Games. Seven semi-structured interviews were conducted with key actors (administrators, coaches, and athletes) who participated in a triple and transversal (local, district and national) Olympic walking training program.

**Results:**

The results provided a better understanding of the program dynamics in the local, regional, and national level, as well as of the short- and long-term effects perceived by the actors of the process on their development, education, health, and career. Recommendations are made for SDP organizations in LAC.

**Discussion:**

Future studies should continue to investigate the SDP initiative in LAC to understand how sport can help development and peace building in this region.

## 1. Introduction

This study contributes to the advancement of the field of Sport for Development and Peace (SDP) research in Latin America and the Caribbean (LAC). Currently, there are very few studies on SDP programs taking place in the LAC region so it is important to better document and understand these experiences and their impacts on participants and the community (1, 2). The present study is the result of a collaborative research between Colombian, Brazilian and Canadian researchers and several stakeholders of sports in Colombia (i.e., athletes and administrators) (3). It aims to describe and understand the perceived effects of a SDP program implemented in Bogota Colombia, and which aimed helping young people from disadvantaged backgrounds in the locality of Ciudad Bolivar. Later, some of these beneficiaries had the opportunity to perform to some of them in Olympic Walking until the highest international level (Beijing 2008, London 2012 and Rio 2016 Olympic Games).

### 1.1. Need for more research on sport for development in Latin America and the Caribbean

SDP projects have increased significantly in recent years around the world (4, 5). In these projects, sport is used as a lever for social inclusion in developing countries, conflict-affected areas, and marginalized or underserved locations in more developed countries. SDP has been defined as, “the intentional use of sport, physical activity and play to achieve specific development goals in low- and middle-income countries and disadvantaged communities in high-income areas” (p.1) (6), which includes, “all forms of physical activity that contribute to physical fitness, mental well-being and social interaction, such as play, recreation, organized or competitive sport, and indigenous sports and games” (p.1) (7). These definitions have since been widely adopted by SDP actors and researchers (8, 9).

However, while these definitions have been accepted by the SDP movement, since the 2003 and 2013 publications there has been increasing variations in the initiatives but also clarification in the terms used to qualify the programs. This has occurred as the initiatives have been studied by researchers. Thus, a majority of authors now refer to them more broadly as S4D or SFD (for Sport for Development), while others persist in including the “peace” in the name and thus using the name more global SDP. While this discussion is still open among researchers, some authors have already proposed more concrete ways to define and operationalize SDP, S4D, SFD, etc. through a conceptual framework named “milestones” (10).

In addition, a literature review provided a comprehensive and holistic picture of the field of SDP research (4). This review revealed that there is a paradox in the geographical contexts among authors and locations of SDP studies. Indeed, although most of the SDP projects are conducted in Africa, Asia, and Latin America, 90% of the research authors are located in North America, Europe, and Australia. Svensson & Woods (5) demonstrated a similar trend in their own mapping of the sector. Overall, empirical research in the field of SDP remains underdeveloped compared to theoretical advances and innovations in other aspects of SDP projects (11, 12). Therefore, more research on SDP programs is needed to better understand their actual contributions to the UN Sustainable Development Goals (SDG), especially in LAC (1, 2, 13).

### 1.2. Sport for development and peace research in Latin America and the Caribbean

LAC is a complex region in which social, economic, and political realities vary widely. Nevertheless, local populations often face daily problems such as violence, crime, lack of access to education, and unemployment. Consequently, sport has become a tool for development and peace in an attempt to respond to current challenges in LAC and research in this area offers several lenses for analysis. Several main areas of research can be identified.

First, some research on SDP in LAC has focused on the use of sport as a value-building tool to combat cultures rooted in patriarchy (gender inequality) and cultural inequalities. Oxford and Spaaij (14–17) ethnographically explored the social, cultural and historical complexities that shape and constrain (gender) space in sport. They show that the social inclusion of young women in traditionally male sport spaces can change the access and configuration of public spaces, especially through the role of “safe space”. However, there are still physical and psychosocial barriers to women's participation that need to be investigated. On the same gender theme, Zipp et al. (18–20) explored the experiences of boys and girls in the *Levelling the playing field program* in the Eastern Caribbean to better understand attitudes related to the role of gender in SDP (human capabilities and lived realities of gender in Caribbean sport). The authors documented how girls and women access and experience sport. Girls' participation is then described as a form of empowerment, even as a mechanism for improving women's quality of life. This is one of the few studies that examine the response of boys vs. girls in sport in LAC. Finally, Hills, Velásquez & Walker (21) discussed sport as an analogy for teaching life skills and redefining moral values through a case study of the *Semilleros de Paz program* in Medellín, Colombia. The study explores how sport has been used to address the legacy of an illegal and violent culture dating back to a time when the city was plagued by drug trafficking and had a reputation as the murder capital of the world. The study documents how Fundación Conconcreto has harnessed the passion of soccer in Colombia in a SDP program, called “*Semilleros de Paz*”*,* as an analogy for teaching life skills and redefining moral values.

Second, the sport for peace axis has also been explored by several publications on SDP in LAC. Cárdenas has documented the mechanisms of using sport for peace (22, 23) and has proposed several papers comparing sport for peace programs in Colombia with Northern Ireland or the Philippines (24, 25). Other authors have also proposed analyses on how sport can be used as a tool for peace (26).

Third, other topics on SDP in LAC have been explored, especially around the evaluation of SDP programs. For example, some authors of the present study have proposed the use of a tool (i.e., *Greimas' actantial model*) to better analyze and understand SDP initiatives at a distance and, in particular, in the context of a SDP program in El Salvador to reclaim public spaces (13). It aimed to strengthen a complex program evaluation work by analyzing organizations' annual reports. The study provides valuable insights into management priorities and practices when the proposed research tool is applied systematically and rigorously. Similarly, Wright et al. (27) investigated the immediate outcomes of an education and training program on 33 youth SDP coaches and their subsequent first-year implementation in Belize. The *Belize Youth Sports Coalition* was a three-year coach exchange project aimed at promoting positive and social development of youth through sport. Wright et al. report that the program was effective in terms of (1) satisfaction with the training, (2) content knowledge, (3) attitudes and beliefs, and (4) ability to apply the content of the educational program. This study also highlights the important relationship between coach education and program implementation. Finally, in response to the real lack of SDP research in this region, Parnell et al. (2) proposed a special issue of the *Journal of Sport for Development* on SDP in the region. In their future considerations, the authors recommend further research on evaluations of SDP programs in LAC to better understand the underlying mechanisms of these interventions. The researchers also suggest examining success stories and best practices in the use of sport as a social development tool in LAC. This will inform and strengthen SDP practice and research internationally. Our study fills this gap by focusing on an SDP program that was developed in Colombia in a suburb of Bogotá and deriving lessons of which could benefit LAC countries as well as other regions.

### 1.3. Colombian context: the *Ciudad Bolívar* district in Bogota

Colombia is a country located in northwestern South America, connecting the south of the continent with Central America. The country has a population of 48,258,494 inhabitants, of which 22.6% (10,906,419) are children under 14 years of age (28). Colombia is currently considered an upper middle-income country with a GDP per capita of over $6,216 (29). However, its income inequality is one of the worst in the world, with a Gini coefficient of 50.3 (30) and with 27% of the population living below the poverty line (31).

The country continues to grapple with a more than 50-year war between the government and illegal armed groups. To date, this war has resulted in more than 220,000 deaths, of which 81.5% are civilians and 18.5% combatants (32). A 2019 UNHCR report indicated that Colombia has 8 million internally displaced persons, making the country the largest number of internally displaced persons in the world (33). Also, according to a national report, more than 2,237,049 children and adolescents are direct victims of the armed conflict (34).

Finally, Bogotá is the cosmopolitan capital of Colombia with more than 7 million inhabitants (28) and its territory is divided into 20 localities. Ciudad Bolívar is one of the urban localities that struggles the most with poverty and violence (35). The locality has a population of 687,923 inhabitants, divided into more than 200 neighborhoods, and is located in the south of the city (36). This locality is known for the high number of displaced people that arrive in the city (37). Many are slum dwellers, and although most of the inhabitants are classified as socioeconomic strata 1 and 2^1^ (38), 14.5% are classified as having “unsatisfied basic needs,” such as inadequate and overcrowded housing, inadequate basic services (e.g., electricity, potable water), and school-age children who do not attend school (36). The locality is considered one of the most dangerous in the city, with very few play spaces for children as well as imminent risk for those left alone, due to the presence of many street gangs and illegal groups (38). Finally, it is the locality in the city of Bogotá with the highest number of children under 5 years of age in poverty (17%) (36).

### 1.4. Community school: Club de Marcha Olímpica on the road to the Olympic Games

This research was established and carried out in collaboration with a SDP non-profit organization, the *Escuela de comunidad* (Community School), created in 1982 in Ciudad Bolivar, and, particularly in relation to its *Club de Marcha Olímpica* (Olympic Walking Club) that was born in 1999. The program used sport (in this case Olympic walking) as a tool to foster the development of young people from low socioeconomic neighborhoods in Ciudad Bolivar^2^. From the beginning, the club received support from the educational institution and the community. The SDP program aimed to help the neighborhood youth build a life program that would lead them away from the difficulties and dangers they face daily (e.g., alcoholism, violence, prostitution, drug abuse, vandalism, and armed groups, among others). The program had two objectives: one academic and one athletic. From the academic point of view, it was intended that the young people would remain in the school system in order to obtain technical or professional training that would enable them to be useful to society and earn a living after their retirement from sports. From the athletic point of view, the program aimed to support young athletes in the process of sports development so that they would perform to the best of their abilities and achieve significant sports results in their category at the district, national and international levels.

Due to the success of the project, in 1999 the *Club Deportivo Isla Potosí* (Isla Potosi Sports Club) was founded, and later joined the Bogota athletics league in order to be able to participate in the inter-league championships in the junior and youth categories. Thus, 8 youngsters between 13 and 16 years old were selected to compete in the 800-, 1,500- and 3,000-meter races. Six of them obtained excellent results and qualified for the National Intercollegiate Games and the South American Games. These results demanded an increasingly organized work that resulted in a rapid increase in the number of young people enrolled in the club. Subsequently, about 100 young athletes began to distinguish themselves in various competitions, gaining the recognition of various media for the school's athletes.

Subsequently, the *Instituto Distrital de Recreación y Deporte* (IDRD, District Institute of Recreation and Sports) of Bogota selected about 40 young people to represent the city in national competitions. The organization supported them with various psychosocial services (e.g., transportation, technical, food, health), which made it possible for about 10 athletes from the club to become national, South American, Pan American and world medalists. After more than 16 years of work, eight athletes from the club have participated in the Olympic Games (Beijing 2008, London 2012, and Rio 2016). During this process, between the difficult reality of their communities of origin and the Olympic Games, most of the athletes gave up their promising sports careers to contribute financially to support their families. Thus, it has been observed that, frequently, young people continue their athletic career up to the age of 18 or 20 years old but have to give up due to the lack of continuous and/or complete support from the State and/or private companies for their preparation and participation in competitions.

### 1.5. Objectives of the study

The lead coordinator of this club, as well as several participants and administrators of this SDP program, asked the research team to rigorously document the process they went through, and the effects observed. Therefore, this study aims to describe and understand the functioning and perceived effects of a sport for development and peace program that was implemented in Colombia to help young people from disadvantaged backgrounds in Ciudad Bolivar, Bogota, to perform in athletics (Olympic walking) at the highest international level (e.g., Beijing 2008, London 2012 and Rio 2016 Olympic Games). More specifically, this study aims to:

1. Describe the process and the structure of the program as perceived by the key stakeholders.
2. Describe the perceived effects that the program had on the development of vulnerable Colombian youth, through the lens of key stakeholders (i.e., athletes, administrator);
3. Understand and analyse the difference between the effects perceived by the two groups of stakeholders who participated in this study, thus identifying the congruences and/or incongruences.

## 2. Methods

### 2.1. Design of the study

A case study methodology was applied, as case studies are well suited to explore complex social, cultural, historical, managerial and procedural phenomena when the situation includes many interesting variables, multiple sources of evidence, and broad theoretical propositions guiding data collection and analysis (39, 40). This study used also a research action design with a collaborative approach (3), and remains qualitative, descriptive, exploratory and inductive; as such, will focus on describing, in detail, the data collected in the interviews analyzed in relation to their context of program implementation. As stated by Creswell (41):

“Qualitative research is emergent rather than strongly prefigured. In the course of a qualitative study several aspects emerge. The research question may change and be refined as the researcher learns what to ask and whom to ask. The data collection process may change as doors for data collection open and close, and as the researcher learns the best places to learn about the central phenomenon of interest. The general theory or model of understanding will emerge from the initial codes, develop into general themes, and coalesce into a grounded theory or general interpretation.” (p.182)

### 2.2. Participants

The participants in this study were key stakeholders (*N* = 7; i.e., participating athletes, coaches, administrators) involved in the SDP program in Bogota (age = 30–60 years). Specifically, they were three participating athletes (1 male and 2 females) who were involved in the program at the local, regional, national and international levels, one coach (1 male) responsible for the implementation of the program at the local (Ciudad Bolivar) and regional (City of Bogotá) levels, and three administrators (1 male and 2 females) responsible for the development of the program at the regional (*n* = 2; IDRD) and national (*n* = 1; Coldeportes) levels. These participants were selected for their involvement as decision-makers or for their role in the implementation of the program, as well as for their availability and willingness to participate in the program and be interviewed. They were recruited on a voluntary basis.

### 2.3. Data collection

Two types of data were used for this research. First, a review of available data on the program was conducted (1 video, 1 written document of 12 pages). The written document was a summary presentation of the program, as well as a presentation of the athletes qualified for international competitions. Second, semi-structured interviews were conducted between February and March 2020 via Skype or Zoom due to the COVID19 pandemic. The interviews were designed to explore key stakeholders' perceptions and experiences of the SDP program at the conceptual level (ideas and intentions) and at the operational level (concrete actions). Questions focused on the short- and long-term outcomes perceived by the athletes and administrators, as well as about the internal processes of the program. Participants were also asked to provide good practices and suggestions for improvement, and some provided additional program documents (e.g., the athlete monitoring and evaluation sheet, the official program set-up document at IDRD). Participants also commented on aspects of the program that did not fit into these categories. The interviews were conducted by TG and NV, took place in a calm and quiet place, were audio-recorded, and lasted approximately 60 min each.

### 2.4. Data analysis

We conducted a combined strategy based on the proposals of Braun & Clarke (42) and Yin (40), which followed seven steps: (a) transcription performed by SV, edited by NV and MG, all Spanish native speakers, (b) familiarization with the data, (c) coding, (d) identification of categories within each theme, (e) review of categories, (f) definition and naming of themes, and (g) redaction. The first step in the data analysis was the verbatim transcription of the interviews. Second, all authors familiarized themselves with the data by listening to the audiotapes and reviewing the transcripts. Third, the data were coded using an inductive process. Fourth, lower-order themes were identified deductively within each main theme. In the fifth and sixth steps, the four authors examined the overarching and lower-order themes, naming and defining them. Finally, the seventh step consisted of disseminating the findings and telling the story from the perspective of the key actors, which is discussed in the results section.

### 2.5. Norms of quality

Quality standards have been applied to ensure the quality of qualitative SDP research (43, 44). Based on these authors, the following strategies were used: (a) breadth, (b) aesthetic merit, (c) worthy subject matter, (d) rich rigor, and (e) transparency. To achieve comprehensiveness (i.e., completeness and quality of evidence), TG and NV conducted interviews and collected data from all key stakeholders. MG, VS and SV provided a detailed description of the data analysis and reported direct quotes from participants so that the reader could judge the quality of the data. TG and NV provided an external point of view. Aesthetic merit (i.e., creative analytic practices) was addressed through a process of inductive thematic analysis, which opened the text to an explanatory interpretation of the information. The study itself is considered a worthy topic, as it was requested by the organization and was relevant, timely and meaningful to their needs. The study demonstrated a high degree of rigor (i.e., use of theoretical concepts, abundance of data, presence of several key stakeholders) by collecting 480 min of interviews. Transparency was achieved through regular discussions among the four authors, coming from three different backgrounds, to understand the data. In addition, bracketing was also used as a quality criterion. Bracketing promotes self-reflection and increases awareness of how personal experiences can affect data collection and interpretation (43, 45). To meet this quality standard, documents (such as background and program description), as well as the authors' field notes were used to better understand the context of the program, its needs, and the key actors involved and their relationships. Finally, the study received ethical approval from the first author's host university committee (3,330_e_2019).

## 3. Results

### 3.1. Stages of the sport for development and peace program

**Figure 1:**
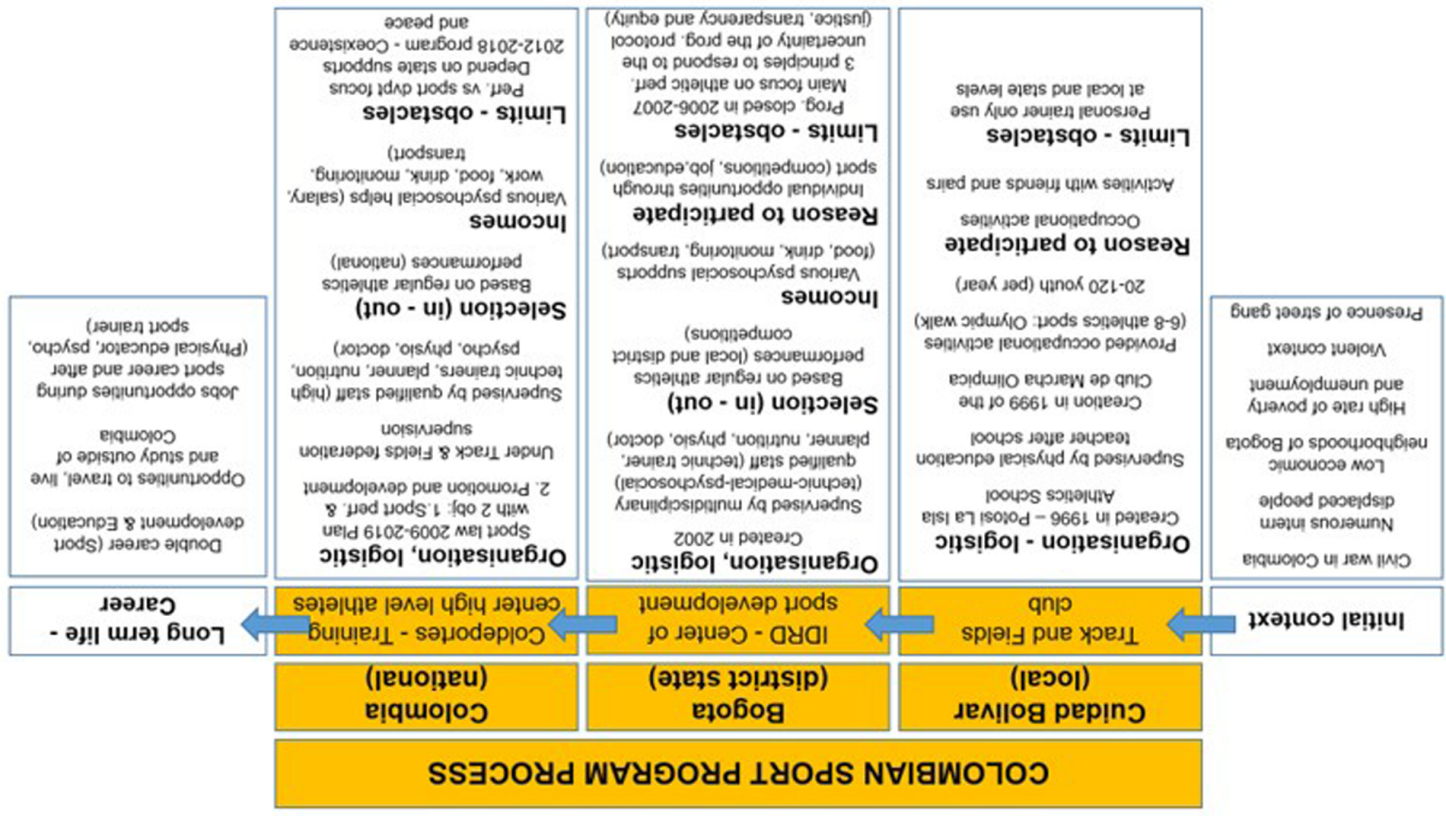
Stages of the SDP program.

Data analysis (i.e., review and key informant interviews) revealed that the SDP program was composed of three stages (Figure 1), each of which corresponded to a different level of sport intervention. The first level, at the Ciudad Bolivar (Local) level, was implemented through the *Club Deportivo Isla Potosí* and physical education classes at the *Cerros del Sur Potosí La Isla*. The second corresponds to the IDRD - *Centro de Desarrollo Deportivo* (Sports Development Center) in Bogota (Regional) and the third and last to the Ministry of Sports, former Coldeportes - *Centro de Entrenamiento de Alto Nivel* (High Level Training Center) in Bogota (National). These three stages are not necessarily linked to each other in a mandatory way. From the IDRD, athletes had to perform, obtain, and maintain good sports results to enter or remain in each of the programs. The programs are therefore part of a temporal process that starts from an initial context (the disadvantaged neighborhood of Ciudad Bolivar) and reaches a long-term trajectory in the person's life (Figure 1).

#### 3.1.1. Stage 1: Club de Marcha Olímpica (*Ciudad Bolívar*)

The first stage of the program was carried out within the framework of the *Escuela de comunidad*, in *Ciudad Bolívar*, in collaboration with the *Escuela de atletismo de Potosí La Isla* since 1996, and then with the *Club de Marcha Olímpica* created in 1999. Conceptually, the club was created by a physical education teacher at the school who intended to help the neighborhood youth build a life program that would lead them away from the daily difficulties and risks they encounter, as previously mentioned. From the beginning, the club received the support of the educational institution and the community. The initial idea was to offer various athletic activities (e.g., running, walking) to attract the maximum number of young people and offer them activities during their free time (e.g., after school, on weekends). The project had two objectives: a training objective and an athletic objective. Firstly, for the young people to continue in the educational system to obtain technical or vocational training that would enable them to earn a living once they had retired from sports. Secondly, the program aimed to support young athletes in a process of athletic development so that they would perform to the best of their ability and achieve significant sports results in their category at the local, district, national and international levels.

#### 3.1.2. Stage 2: Instituto Distrital de Recreación y Deporte (IDRD - distrito de Bogotá)

The second stage of the program was taken over by the IDRD of Bogota, which created a program to support young sports talent in the region between 2002 and 2007. Conceptually, this program was developed by a multidisciplinary IDRD team (i.e., management, psychosocial team, sports science team and technical team), focusing on talent identification while giving an opportunity to young people from vulnerable communities.

The program offered multidisciplinary (i.e., technical, medical, and psychosocial) support to each athlete. Athletes were recruited and selected based on their performance in local and district competitions. The permanence in the program depended on permanence criteria and their solid performance. Unfortunately, this program was closed in 2006–07 for two main reasons. First, the focus was mainly on sports results, neglecting support for meeting psychosocial needs for young athletes (e.g., financial aid, food assistance, social assistance). Second, the official administration decided not renew the funding of this project.

#### 3.1.3. Stage 3: ministry of sports (former Coldeportes) (Bogotá, Colombia)

The third stage of the program was integrated into the training and high-performance center for athletes of the institution responsible for sports in Colombia, the former Coldeportes, located in Bogotá. Conceptually, this program was part of the *Plan Decenal del Deporte, la Recreación, la Educación Física y la Actividad Física 2009–2019* (Sports Law and the Ten-Year Plan for Sports, Recreation, Physical Education and Physical Activity 2009–2019). The plan had two axes: (a) performance sport and (b) promotion and development through sport. Like IDRD, the objective was to select and provide technical, medical, and psychosocial support to athletes representing Colombia in international competitions (e.g., Pan American Games, World Championships, Olympic Games).

The program was attached to the Colombian Athletics Federation and under the supervision of its qualified members. Several services were offered to athletes belonging to Coldeportes, such as technical support (e.g., high-level training, performance monitoring and evaluation), logistical support (e.g., transportation, accommodation), economic support (e.g., salary, work), nutritional support (e.g., food, beverages), psychosocial and medical support (e.g., psychology, physiology, medicine), and mediatical support (e.g., communications, public and private relations). Again, these psychosocial services were aimed at enabling athletes to study or train for further development outside of their sporting career. As in the IDRD, athletes were selected on the basis of their results in regional and national competitions. The better they did, the more hope they had of continuing in the program.

### 3.2. Perceived effects of the sport for development and peace program by key stakeholders

We present the results of the perceived effects of the SDP program separately for athletes and administrators. Athletes were the beneficiaries of the program, while administrators were those who conceptualized (partially or fully) and implemented the program. It was not easy to identify how the IDRD are pursuing social development and peace for example. On this, the three sections of the program (Club de marcha, IDRD and Coldeportes) were not thought/developed in relation to each other, but in independent ways as their structures. This study aims (obj1) to trace globally the experience of the youth through these three programs and thus the overall functioning of the process, but not to explain how IDRD, for example, aimed to pursue development and peace, other than the elements we mention on p. 9–10, Section 3.2.1 and 3.2.2.

#### 3.2.1. Effects of the program as perceived by athletes

**Figure 2:**
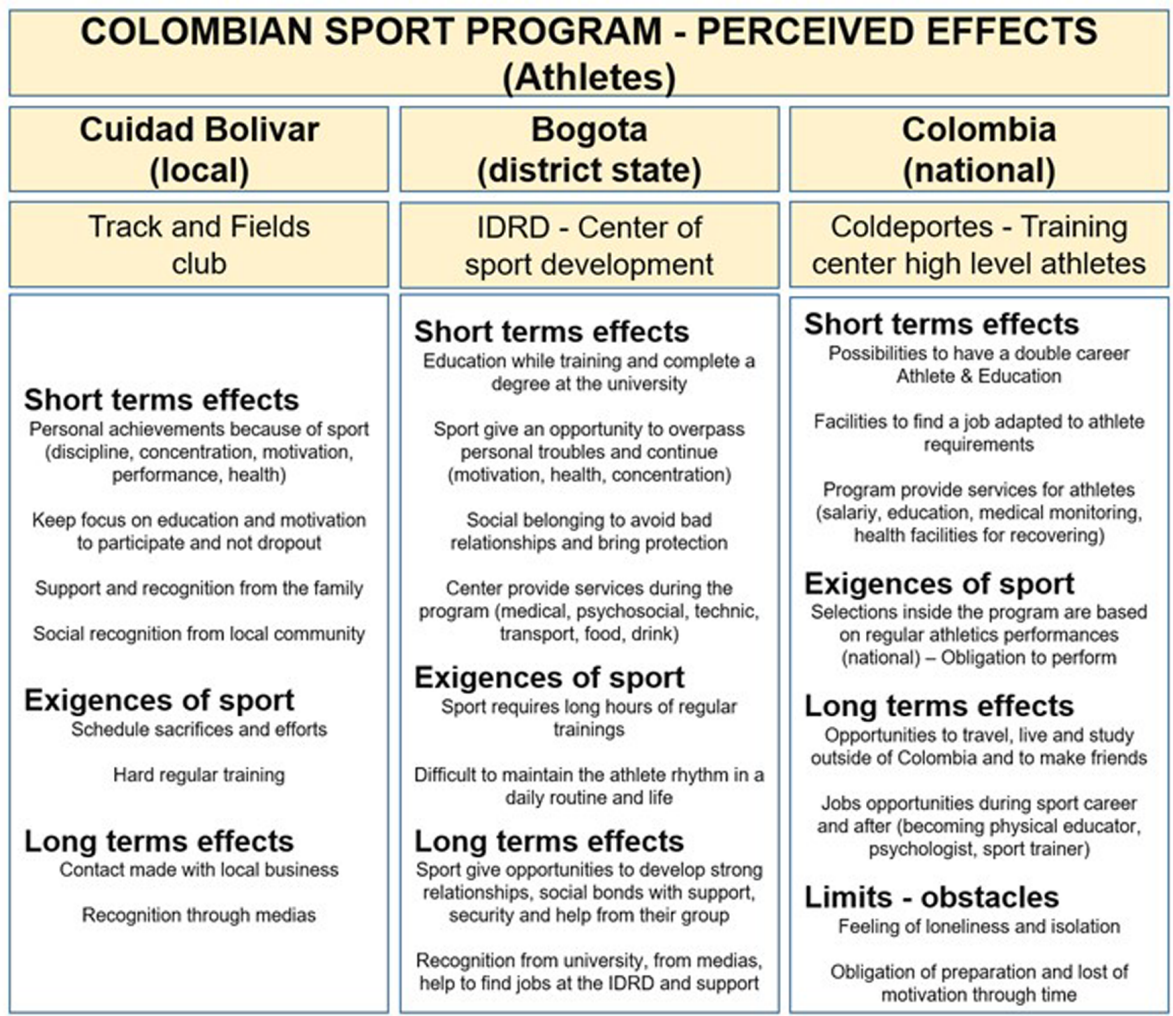
Perceived effects of the SDP program by athletes.

In general, the athletes reported perceiving the short- and long-term effects and demands of the sport (Figure 2). All of them also report that the sport has been very demanding as they have gone through the three stages of the SDP program.

For stage 1 and in the short term, they mention that the sports program allowed them to achieve several personal goals on a day-to-day basis (e.g., maintaining discipline, staying focused, being motivated, performing well, maintaining their health), while maintaining their focus on education and their motivation to participate and not drop out of the project. The program also appears to have provided them with social support and recognition from their family and community. In the long term, they claim to have gained recognition from local media that covered their performances, as well as a network of contacts that will help them integrate professionally and economically. Of course, athletes also mention the high demands of the sport and the many sacrifices or efforts they had to make during the program to achieve success. For example, they mention the very tight schedule between studies and the sports program, or a very busy and therefore very exhausting training schedule.

For stage 2 and in the short term, we found the same elements as above. Athletes also mention that the sports program helped them to overcome individual obstacles in their daily life (e.g., personal problems, lack of motivation or concentration) but also to avoid bad situations, while providing them with social protection. They also report receiving a range of services from IDRD (e.g., access to a doctor, psychosocial monitoring and assessment, technical supervision by qualified personnel, food resources, transportation). In the long term, the program has provided them with the opportunity to develop strong relationships with their peers, who have often become lifelong friends as well. The program has also helped them receive support from their group and develop a sense of security within the group. Despite the increasing demands of the sport, the program has allowed the athletes to receive strong recognition from local universities (who have supported and taken ownership of their study project with financial support and training flexibilities), from the media (who have helped them find jobs and be recognized by their community) as well as from the IDRD (to find jobs that fit with their sporting career).

Finally, in phase 3, athletes reported having the opportunity in the short term to pursue a dual career (athlete and student), help in finding a job that suits their sporting requirements, and an extensive list of support services. Athletes place great emphasis on the demands of sport in relation to selections and the pressure to maintain high performance and results. In the long term, athletes report having had the opportunity to travel for national and international competitions, to make truly good friends for life, and the opportunity to access quality, skilled employment as a result of their success at university. They became physical education teachers, psychologists, coaches, and entrepreneurs. Finally, many athletes regret the feeling of isolation and loneliness in the field of high performance, and in particular the fact that they could not be accompanied by their coach from the first program, in whom they had full confidence. These factors explain the loss of motivation and interest in competitive Olympic walking in the long term.

#### 3.2.2. Effects of the program as perceived and intended by the administrators

**Figure 3:**
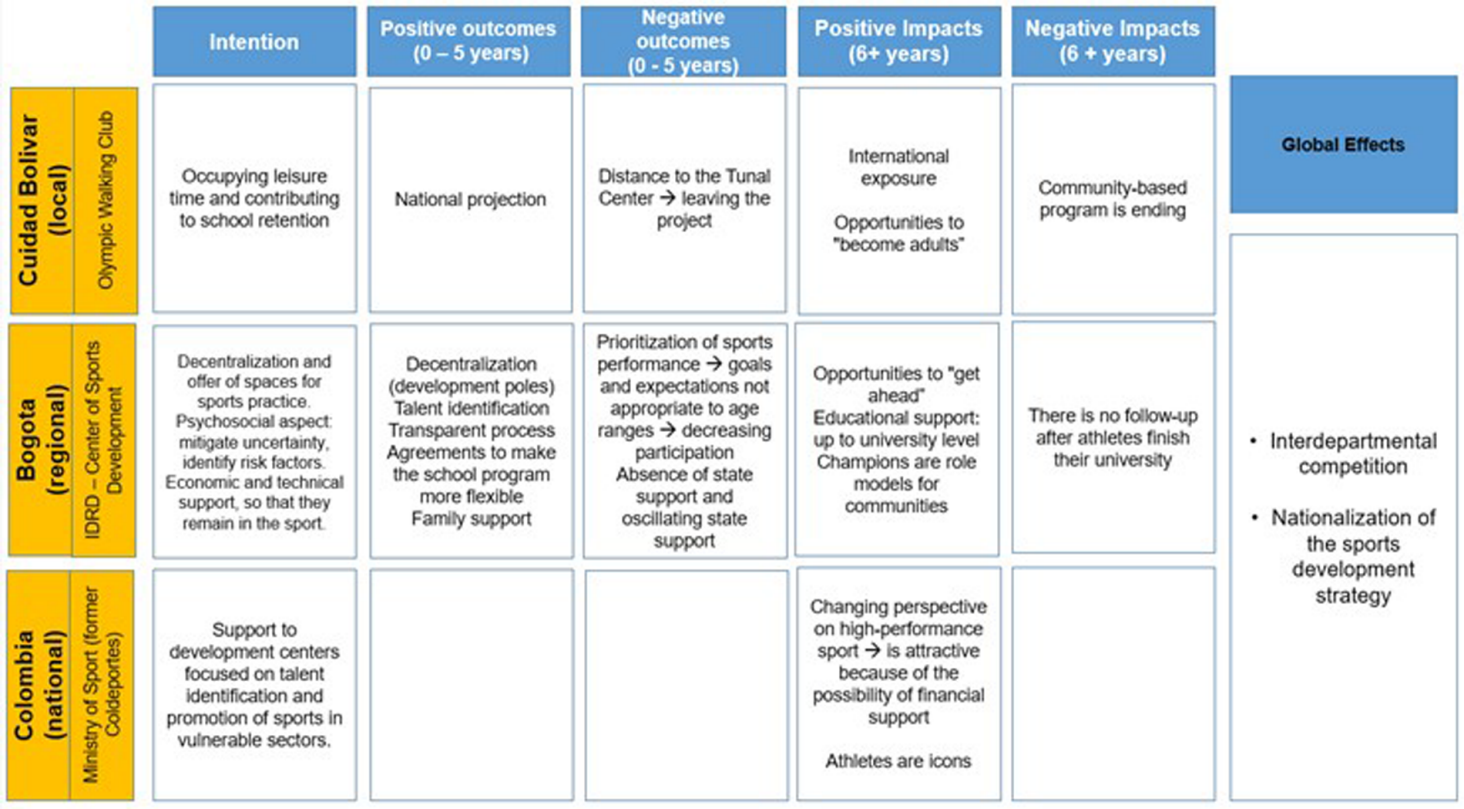
Perceived and intended effects of the SDP program by administrators.

As expected, the administrators provided their perspectives through more “managerial” lens, that is, with a great focus to the process that was implemented and its shorter-term outcomes and long-term impacts. In general, their perceptions are very much in line with the way the program has worked (see previous sections) at different stages (Figure 3). They all commented in how proud they are of the structures put in place and mention many of the effects perceived by athletes at different stages of the program. However, several administrators regretted not being able to pursue the sport-for-development component throughout all of the stages to the detriment of athletic performance.

The administrators mention that the three stages were structured little by little in view of the success of the Ciudad Bolivar Club and then the IDRD. At the local level, the initial intention of occupying youth's free time with valuable activities has been accomplished in the short and long term, giving the athletes not only the opportunity of “becoming adults” in an extremely violent community, but also reach national and international projection. Nevertheless, the further institutionalization of the community programme translated into a deeper focus on performance and athletic results. For example, the training grounds of the *Club de Marcha Olímpica* was transferred from Ciudad Bolívar to the more advanced centre in the neighbourhood of *Tunal*. The distance, although not big, prevented more vulnerable children and young people to commute and take part in the practice sessions. As a result, the community programme as once idealized by its founders is reaching an unsustainable stage, loosing its community-target foundation.

If on the one hand the IDRD was attracted by the success of the *Club de Marcha Olímpica* in Ciudad Bolívar, the organization was also motivated to further invest in sport in the city of Bogotá due the existing athletic rivalry among other Colombian departments. All administrators mentioned the importance of the national school tournaments for the sport environment in the country. Bogotá's SFD strategy intended, then, to decentralize the offer of spaces for sports practice, enlarging the basis of the pyramid of sports talents and reaching out to underprivileged and hard-to-access communities.

Before the establishment of the project at IDRD, it was necessary an extensive investigation of the real psychosocial conditions of these communities. They identified not only the need of offering economic and technical support, but also of establishing a transparent process for athletes to reach higher levels in their sports career. This was essential to mitigate the uncertainty of the sports career and motivate youth to remain in sports programmes. The multidisciplinary team was also key in reaching agreements with other key sectors (i.e., educational institutions, transportation companies). In the long term, this support is perceived to have contributed to youth reaching to university-level education and becoming role models in their own communities.

The model of SFD implemented in Bogotá attracted the attention of other departments, as well as of Coldeportes, currently the Ministry of Sports. The nationalization of the strategy is promoted and implemented by the Ministry, which supports the establishment of development centers focused on talent identification and sports promotion in vulnerable sectors across the country. The possibility of progressive financial support if an athlete reaches higher levels of sports performance became an attractive alternative for vulnerable youth to support their families and represent their communities. According to one of the interviewees, the perspective on high-performance sports has changed to a more positive one.

However, the prioritisation of sports results is considered the main negative outcome by all the participants in this study. Children and youth were expected to reach unappropriated goals and standards for their age groups related the high-level expectations. The higher the demand from their coaches or clubs, the higher the rate of dropping out of sports programmes. They also report that most of the athletes gave up their promising sports careers to contribute financially to support their families. Thus, they note that young people become accomplished athletes at 18–20 years of age but have to give up because of the lack of continuous and comprehensive support from the state or private companies for their preparation and participation in competitions. The oscillating support from the state is considered relevant for the sustainability of the whole process. Sports policy remains a government policy, that is depending on the elected representatives, rather than a state policy that is stable and ongoing.

## 4. Discussion

### 4.1. Major findings

With respect to the operation of the SDP program, it is noted that the program was developed over a long-term process of more than 10 years, in which three successive and complementary programs were established, each belonging to a different structure (i.e., local, district, national). Two athletes interviewed went through stages 1–2–3, while one only participated in 1–2. As for the administrators, 1 participated in stage 1, 2 worked at IDRD in stage 2 and 1 in stage 3 at the Ministry of Sports. The results of our study show that the three programs had two objectives related to performance and development. However, over time, IDRD and Ministry of Sports prioritized the performance component of the athletes to the detriment of the psychosocial support service structure. As a result, the number of athletes who have gone far in the program process has been drastically reduced (*n* = 8 athletes), according to the summary document and comments reported in the interviews. The result is a pyramidal structure with a broad base involving many young people, but a narrow tip that would have been accessible to a larger number of athletes if a balance had been maintained between the initial objectives of the program.

Regarding the perceived effects of the SDP program, key stakeholders reported short- and long-term effects of the sports program, both sporting and educational, for both athletes and administrators. In this regard, we observed that the effects perceived and expected by the administrators largely correspond to the effects experienced by the athletes during the program, however they stated regretting not having been able to continue or further enrich the sport for development component considering the great result observed in the community and in the participants, since the performance component was the one subsequently emphasized by IDRD and Coldeportes. Therefore, this program initially conceived to occupy young people from disadvantaged areas of Bogota and Ciudad Bolivar in particular, actually fulfilled its function of democratization and facilitating access to training and education in stages 1 and 2 but was later distorted. Obtaining competitive results and athlete performance was, in fact, the main motive of this program, especially for stage 3. Although our study demonstrated the existence of three independent stages in the aforementioned SDP program, athletes often recounted their experiences as a single process. Thus, the transitions between stages appeared to be well articulated, as long as they met the requirements of selection and maintenance of successful outcomes by the athletes. At this point, it is important to remember that the program is read by administrators and athletes as a long-term holistic process for development. Let us not forget that these programs are rooted in the reality of a complex initial context with the long-term perspective of leading a dual career (sport and education).

### 4.2. Perspectives on Latin American and Caribes literary research

This study adds to the sparse record of SDP research conducted in LAC (2, 4, 5). It has documented how three programs using SDP have been articulated in a more holistic process of training and coaching athletes, something that has not been done before. Three elements seem relevant to discuss here.

First, this study is quite unique in that it is unlike any previously conducted on SDP in LAC. Certainly, commonalities can be established with research that has documented the experiences of participants in SDP programs (14, 19), but its subject matter remains original as do the official organizations (Ciudad Bolivar, IDRD, Ministry of Sports) that hosted its stages. In our opinion, it is an interesting illustration of a collaborative model that can be built at various levels (local, provincial, national) and that involves various actors (e.g., school, civil club, municipality, state, country) as recommended by Parnell et al. (2) in their future considerations. Furthermore, this study shows that one of the main causes of program termination was the disregard of the initial objectives of the program (sport for development and development of sport) by the new leadership of the governmental institutes that financially and technically sustained the program, focusing on the performance objectives as the sole condition for maintaining support. This demonstrates once again that one of the biggest challenges for sport organizations in developing countries is the gap in terms of sport governance and policy, in how sport organizations and systems are run and controlled, and who is at the helm of program management, which has become a central concern of sport management in recent years (46–48). The results of this research may also constitute a first level of evaluation of the SDP program and would deserve to be deepened in a multidisciplinary, pre-post, and quantitative comparison perspective as some studies have begun to do (27).

Second, a more detailed exploration of this program using sport for development leads us to ask: is this program really an SDP project and on what basis? Following the guidelines proposed by Gadais et al. (49), we consider that this program falls within the SDP sector as the objectives (*because*) that motivated the launch of this program are in line with several of the United Nations Sustainable Development Goals (e.g., SDG1, SDG2, SDG2, SDG3, SDG4, SDG8, SDG10) (49). Obviously, much emphasis has been placed on the goal of sports performance, but the analyses of the conceptual and organizational functioning confirm the authors' proposals to fight poverty, reduce hunger, have optimal health, provide quality education, etc. This study also demonstrates that sport programs can help promote greater employability of vulnerable groups and thus contribute to the economic growth of society. Finally, it could be led to think that these programs individually or associated are in line with the theories of Youth Positive Development (50) or the Lifeskills theory (51). However, these approaches don't fit our subject because they have been developed in Global North countries specific contexts for the development of athletes or students through sport (52). Therefore, those theories are not useful to understand our programs because elements are misaligned with the specificities of the LAC region. Consequently, none of those connect with the intention of the administrators of our studied programs as well as the context of Colombia. Thus, these theoretical frameworks propose a very Anglo-Saxon view of the scientific literature and program vision that does not resonate with the Latin American context, nor with what administrators have attempted to do in these programs in Colombia. Thus, it does not seem appropriate to draw on work from these fields to enrich the discussion.

Third, we believe that this project is a good example of peacebuilding through sport. In all three programs, sport has been used (stages 1 and 2 especially) to promote peacebuilding, but also to connect people or communities that have been affected by the conflict (22). In our opinion, this study reveals new mechanisms for the use of sport for peace (22, 23, 26) through a sport performance objective. This SDP program process allowed to engage (*Hook*), divert (*Diversion*), entertain and integrate (*Integration*) youth through Olympic marching. It would be pertinent to compare the results of this study with the work of other organizations in international SDP contexts.

Finally, literature reviews conducted on SDP (4, 5) had mainly identified soccer as the sport activity preferred by the projects (in 90% of the cases). In this program, the physical activity used as a support was Olympic walking, which is a bit particular as it does not correspond to any Colombian or LAC cultural standard, where soccer remains the emblematic activity par excellence as an attractive referent for young people. However, nowadays in Colombia, when talking about Race Walking, this sector of the country and the city is referred to as a mandatory place to train with the best in the country, due to the technical development it has achieved and the favorable altitude conditions for the modality, taking into account that the city of Bogota is located at 2,640 meters above sea level.

In this sense, this activity could be interesting for future projects due to its low cost and the fact that it does not require sophisticated facilities or equipment for its implementation, which is especially interesting in the context of the DDP. In addition, this program continues to count on the coach who started the walking school in the sector and who today is one of the coaches responsible for the athletics and Olympic walking section of the IDRD. Finally, it is symbolic, since one of the spaces used for training is a place steeped in history. In fact, the club used to train daily near a tree called “*el palo del ahorcado*”, where several people were hanged during clashes between street gangs and drug traffickers. A tree and a place highly charged with symbolism for the entire community of Ciudad Bolivar.

### 4.3. Limits

This study has two limitations. First, few athletes make up the corpus of participants in this project. Despite having sufficient documentation to support the conception and objectives of the program, it would be desirable to be able to complete the results of this research with a larger sample of participants, including some who only participated in phase 1 of the project. Increasing the N would also allow us to confirm the trend of our results and possibly attempt to further quantify their effects. The second limitation is related to the COVID-19 pandemic, which modified the initial protocol of our study and, consequently, field observation had to be eliminated. This element missing in the triangulation of the data could made lost precision in the interpretation of the data, however, we make sure Colombians partners can comment and add on the data interpretation.

### 4.4. Recommendations

This project also allows us to propose several recommendations related to the results obtained and the SDP projects in LAC. Regarding the implementation of SDP projects in LAC:

- Invite district and national authorities in Colombia, but also in other LAC countries, to integrate a development component in their sports policies and programs;
- Promote multidisciplinary and multisectoral collaboration in the development and implementation of SDP programs;
- Elaborate long-term development programs that allow for the development of dual careers: academic and sports;
- Reserve in the management plans of the cities or the country, the necessary resources to financially support this type of programs in coordination with the educational system;
- Create efficient mechanisms for the dissemination of the processes and results of this type of initiatives and their impact on society;
- Define criteria for the selection of personnel responsible for sports programs that recognize the ideal competencies for the positions offered.

In relation to scientific research:

- Enrich research data through collaborative meetings with key players in the sector. The aim is to enrich the quality of the results, but also to confront the results obtained with the perceptions of the stakeholders of this SDP.
- Ensure respect for the international law of all children and adolescents, including the right to equal education and to have an inclusive education that respects the developmental needs of the child;
- Ensure that the overall development of children, including physical and mental health (i.e., enjoyment), is promoted and not only certain aspects (e.g., physical fitness);
- Verify the learning environment to promote quality education;
- Carry out psychological and social follow-up in relation to potential trauma, brainwashing and forms of indoctrination of children and adolescents and promote their harmonious reintegration into society, potential gaps with families and communities;
- Be attentive to the lack of autonomy, creativity, participation and critical thinking skills of children who have been affected by armed conflict;
- Address the lack of fulfillment of basic physical as well as basic psychological needs of the youth;
- Verify the teaching conditions and accompany the teachers in training and pedagogical monitoring in order to provide quality education for all.

## 5. Conclusion

This study contributes to the advancement of the field of SDP research in LAC. It is the result of a collaborative research that aimed to describe the experiences and perceptions of Colombian youth during a SDP program that led some of them, belonging to disadvantaged neighborhoods of Bogotá, to participation in the discipline of Olympic Walking in Olympic Games. The results provided a better understanding of the organization and functioning of the separate, but also combined programs of the Ciudad Bolivar Community School, the IDRD of Bogota and Coldeportes. Our study also revealed the perceived effects of these programs by participating athletes and administrators. In particular, the short- and long-term effects of the process for Colombian youth on their development, education, health, and professional careers. We also proposed recommendations to official LAC SDP organizations and researchers in the field, so that other initiatives can learn from this example of a SDP program in Colombia and its effects on participants.

Following the proposals of Cardenas (25) and Parnell et al. (2), we will encourage researchers to continue scientific research in the LAC region in relation to SDP. Given the complexity and diversity of the context, more studies are needed to understand how sport can contribute to development and peacebuilding in this region. Therefore, it is relevant to explore grassroots mobilization and sustainable development initiatives in response to specific social concerns. We recommend that research on the evaluation of SDP programs be intensified, including close collaboration between academic institutions, community-based organizations, donors, and the international community. Finally, future research should also focus on successful experiences and best practices in the use of sport as a tool for social development in LAC, to enrich SDP research and practice globally.

## Data Availability

The raw data supporting the conclusions of this article will be made available by the authors, without undue reservation.

## Footnotes

^1^In Colombia, a socio-economic stratification system classifies urban populations into different strata with similar economic characteristics on a scale from 1 to 6 with 1 as the lowest income area and 6 as the highest. The system is organized so that the people from upper layers (strata 5 and 6) pay more for services like electricity, water and sewage than the groups in the lower strata.

^2^ https://youtu.be/yExAoWig8jA
